# Mediport use as an acceptable standard for CAR T cell infusion

**DOI:** 10.3389/fimmu.2023.1239132

**Published:** 2023-10-27

**Authors:** Maya Eylon, Snehit Prabhu, Samuel John, Maxwell J. M. King, Dhruv Bhatt, Kevin J. Curran, Courtney Erickson, Nicole A. Karras, Christine L. Phillips, Prakash Satwani, Michelle Hermiston, Erica Southworth, Susanne H. C. Baumeister, Julie-An Talano, Margaret L. MacMillan, Jenna Rossoff, Challice L. Bonifant, Gary Doug Myers, Rayne H. Rouce, Keri Toner, Timothy A. Driscoll, Emmanuel Katsanis, Dana B. Salzberg, Deborah Schiff, Satiro N. De Oliveira, Christian M. Capitini, Holly L. Pacenta, Thomas Pfeiffer, Niketa C. Shah, Van Huynh, Jodi L. Skiles, Ellen Fraint, Kevin O. McNerney, Troy C. Quigg, Joerg Krueger, John A. Ligon, Vanessa A. Fabrizio, Christina Baggott, Theodore W. Laetsch, Liora M. Schultz

**Affiliations:** ^1^ College of Medicine, Central Michigan University, Mount Pleasant, MI, United States; ^2^ Department of Pediatrics, Division of Hematology and Oncology, Stanford University School of Medicine, Palo Alto, CA, United States; ^3^ Department of Pediatrics, The University of Texas Southwestern Medical Center/Children’s Health, Dallas, TX, United States; ^4^ Department for Biology, Stanford University, Palo Alto, CA, United States; ^5^ Department of Pediatrics, Memorial Sloan Kettering Cancer Center, New York, NY, United States; ^6^ Department of Pediatrics, City of Hope National Medical Center, Duarte, CA, United States; ^7^ Department of Pediatrics, University of Cincinnati, Cincinnati, OH, United States; ^8^ Cincinnati Children’s Hospital Medical Center, Cancer and Blood Disease Institute, Cincinnati, OH, United States; ^9^ Division of Pediatric Hematology, Oncology and Stem Cell Transplant, Department of Pediatrics, Columbia University Medical Center, New York, NY, United States; ^10^ University of California, San Francisco Benioff Children’s Hospital, San Francisco, CA, United States; ^11^ Dana-Farber/Boston Children’s Cancer and Blood Disorders Center, Dana Farber/Boston Children’s Hospital, Boston, MA, United States; ^12^ Department of Pediatric Hematology Oncology, Medical College of Wisconsin, Milwaukee, WI, United States; ^13^ Department of Pediatrics, Division of Pediatric Blood and Marrow Transplantation, University of Minnesota Medical School, Minneapolis, MN, United States; ^14^ Division of Pediatric Hematology, Oncology and Stem Cell Transplantation, Ann & Robert H. Lurie Children’s Hospital of Chicago, Chicago, IL, United States; ^15^ Sidney Kimmel Comprehensive Cancer Center, Division of Pediatric Oncology, Philadelphia, MD, United States; ^16^ Children’s Mercy Hospital, University of Missouri, Columbia, MO, United States; ^17^ Bone Marrow Transplant/Stem Cell Transplant Program, Texas Children’s Cancer Center, Houston, TX, United States; ^18^ Division of Blood and Marrow Transplant and CAR-T Program, Children’s National Hospital, Northwest, DC, United States; ^19^ Pediatric Transplant and Cellular Therapy, Duke Children’s Hospital & Health Center, Durham, NC, United States; ^20^ Department of Pediatrics, University of Arizona, AZ, United States; ^21^ Center for Cancer and Blood Disorder, Phoenix Children’s Hospital, Phoenix, AZ, United States; ^22^ Division of Hematology/Oncology, Rady Children’s Hospital, San Diego, CA, United States; ^23^ Department of Pediatrics, University of California Los Angeles (UCLA) Mattel Children’s Hospital, Los Angeles, CA, United States; ^24^ Department of Pediatrics and Carbone Cancer Center, University of Wisconsin School of Medicine and Public Health, Madison, WI, United States; ^25^ Cook Children's Hematology and Oncology, Cook Children’s Hospital, Fort Worth, TX, United States; ^26^ Department of Pediatrics, Department of Pediatrics, Children’s Hospital of Philadelphia, University of Pennsylvania, Philadelphia, PA, United States; ^27^ Saint Louis Children’s Hospital One Children’s Pl, Saint Louis, MO, United States; ^28^ Yale Medicine, Yale University and Yale New Haven Children’s Hospital New Haven, New Haven, CT, United States; ^29^ Pediatric Oncology, CHOC Children’s Hospital of Orange County, Orange County, CA, United States; ^30^ Riley Children Health, Indiana University Health, IN, United States; ^31^ Division of Pediatric Hematology, Oncology, and Cellular Therapy, The Children’s Hospital at Montefiore, Bronx, NY, United States; ^32^ Department of Pediatrics, John Hopkins All Children’s Hospital, St. Petersburg, FL, United States; ^33^ Section of Pediatric BMT and Cellular Therapy, Helen DeVos Children’s Hospital, Grand Rapids, MI, United States; ^34^ Division of Hematology/Oncology, The Hospital For Sick Children, Toronto, ON, Canada; ^35^ Health Pediatric Blood & Marrow Transplant and Cellular Therapy, University of Florida, Gainesville, FL, United States; ^36^ Colorado Children’s Hospital, University of Colorado, Boulder, CO, United States

**Keywords:** chimeric antigen receptor T cell, immunotherapy, cancer, immune cell engineering, mediport, implanted catheter

## Abstract

**Introduction:**

Mediport use as a clinical option for the administration of chimeric antigen receptor T cell (CAR T cell) therapy in patients with B-cell malignancies has yet to be standardized. Concern for mediport dislodgement, cell infiltration, and ineffective therapy delivery to systemic circulation has resulted in variable practice with intravenous administration of CAR T cell therapy. With CAR T cell commercialization, it is important to establish practice standards for CAR T cell delivery. We conducted a study to establish usage patterns of mediports in the clinical setting and provide a standard of care recommendation for mediport use as an acceptable form of access for CAR T cell infusions.

**Methods:**

In this retrospective cohort study, data on mediport use and infiltration rate was collected from a survey across 34 medical centers in the Pediatric Real-World CAR Consortium, capturing 504 CAR T cell infusion routes across 489 patients. Data represents the largest, and to our knowledge sole, report on clinical CAR T cell infusion practice patterns since FDA approval and CAR T cell commercialization in 2017.

**Results:**

Across 34 sites, all reported tunneled central venous catheters, including Broviac^®^ and Hickman^®^ catheters, as accepted standard venous options for CAR T cell infusion. Use of mediports as a standard clinical practice was reported in 29 of 34 sites (85%). Of 489 evaluable patients with reported route of CAR T cell infusion, 184 patients were infused using mediports, with no reported incidences of CAR T cell infiltration.

**Discussion/Conclusion:**

Based on current clinical practice, mediports are a commonly utilized form of access for CAR T cell therapy administration. These findings support the safe practice of mediport usage as an accepted standard line option for CAR T cell infusion.

## Introduction

Chimeric antigen receptor T cell (CAR T cell) therapy is an approach to adoptive cell therapy that has reformed management of relapsed and refractory B-cell acute lymphoblastic leukemia (B-ALL) in children and young adults. Clinical studies using CAR T cell targeting the canonical B cell marker, CD19, have demonstrated complete remission rates of 70-90% in B-ALL, driving FDA approval for patients <26 years with refractory B-ALL or disease in ≥2^nd^ relapse (1–5).

CAR T cell products are personalized therapies whose production requires leukapheresis to procure a T cell product, followed by T cell activation, then CAR integration via genomic engineering, most commonly achieved in the clinical setting using lentiviral or retroviral transduction (2). CAR T cells are then expanded ex vivo in the presence of cytokines prior to patient infusion (6). Commercial CAR T cell production is highly specialized and extremely costly, nearing half a million dollars per patient (7). Additionally, the therapeutic window for cell infusion is limited. Due to the complex nature of CAR T cell manufacturing and the high-risk patient population, it is vital to ensure successful systemic delivery of the clinical CAR T cell product and avoid clinical processes that could risk compromising the product.

Currently, there is no standard recommendation for acceptable venous access infusion for the administration of CAR T cell therapy. Current options for infusion include tunneled venous catheters (Broviacs^®^ and Hickmans^®^), mediports, peripherally inserted central catheter (PICC), and peripheral intravenous (IV) catheters (8). Tunneled venous catheters with external line access, such as Broviacs^®^ and Hickmans^®^, are functional line options, but in clinical practice are more often placed for allogeneic hematopoietic stem cell transplantation (HSCT) and are not usually present in patients receiving upfront leukemia therapy or CAR T cell therapy. While PICC lines can be placed for the administration of CAR T cell therapy, PICCs have demonstrated significant risk of complications, including central-line associated infections, catheter occlusion, and thrombosis (deep vein thrombosis and pulmonary embolism) (9).

Mediports are implanted venous access ports, typically placed under the skin on the upper chest, used to administer medications, fluids, blood products, and chemotherapy. Benefits of mediports include their ease of access, decreased risk of infection compared to tunneled venous catheters, and improved quality of life (10–12) (13). Challenges associated with mediports include risk of thrombosis, subcutaneous port movement, port blockage, and difficulty of device usage in young children (8). Mediports are commonly placed for management of upfront leukemia and although not always present at relapse, they are often present in patients who meet indications for clinical CAR T cell therapy. While there are many benefits to mediports, due to the highly personalized and expensive nature of CAR T cell therapy there is a theoretical concern of infiltration. HSCT serves as the paradigmatic example of adoptive cellular transfer, and common pediatric practice is to avoid mediport use for HSCT cell infusions, to ensure more direct systemic stem cell delivery (14, 15). Mediport infiltration can occur if the implanted needle dislodges from the port, which could result in the therapy entering the extravascular space. Further concern is that the loss of therapy may go unnoticed because the port is subcutaneous. These theoretical concerns have resulted in variable practice in line administration in real world settings, as practitioners debate the relative risks and benefits of placing new PICC lines or utilizing existing mediports for CAR T cell infusion.

In this study we conducted a survey of medical centers in the Pediatric Real-World CAR Consortium (PRWCC) to establish usage patterns of mediports in the clinical commercial CAR T cell setting to provide an evidence-based standard of care recommendation for mediport use for CAR T cell infusions.

## Methods

In this retrospective cohort study data on the usage of mediports and occurrence of infiltration was collected from a two-tiered survey distributed to the thirty-four medical centers in the PRWCC. The first survey collected aggregate data at the site level on the method(s) of venous access used for CAR T cell therapy infusion in clinical practice (**Supplemental Figure 1**). Included was reporting on incidence of infiltration for sites that used mediports and peripheral intravenous devices for administration. Incidence of infiltration was retrospectively reported based on clinical assessments at PRWCC centers via patient, nursing and/or physician reporting, and clinical documentation of surrounding edema, erythema, and/or pain at the mediport site during and after the infusion administration.

The second survey collected patient specific data from individual sites on the total number of patients treated with CAR T cell therapy within the study timeframe, as well as the form of venous access utilized for each infusion (**Supplemental Figure 2**). Data represents clinical practice patterns over the first five years of commercial CAR T cell therapy usage since FDA approval in August 2017 through September 1^st^, 2022.

## Results

In this study, retrospective data were collected across 489 patients from 34 PRWCC sites. A two-tiered survey approach was applied (**Table 1**). The first tier of the survey included four questions regarding the methods of venous access utilized in each medical center for CAR T cell therapy administration (**Supplemental Figure 1**) The survey specifically inquired about the use of tunneled central venous catheters, peripherally inserted central catheters, mediport central venous catheters, and peripheral intravenous catheters. For centers using mediports and peripheral intravenous catheters, there was a follow-up question regarding the number of incidences of therapy infiltration. The second tier of the survey collected data on total number of Tisagenlecleucel infused patients between August 2017 – September 1^st^, 2022, as well as the route of administration for each infusion (**Supplemental Figure 2**).

**Table 1:** PRWCC medical centers surveyed on line utilization for clinical CART delivery (N = 34).

Ann & Robert H. Lurie Children’s Hospital of Chicago	Medical College of Wisconsin
Children’s Health Orange County	Memorial Sloan Kettering Cancer Center
Children’s Hospital at Montefiore	Phoenix Children’s Hospital
Children’s Hospital of Philadelphia	Rady Children’s Hospital San Diego
Children’s Mercy Hospital (Kansas)	Riley Children Health, Indiana University Health
Children’s National Hospital	St. Louis Children’s Hospital
Cincinnati Children’s Medical Center	Texas Children’s Cancer Center
City of Hope National Medical Center	UCSF Benioff Children’s Hospital
Columbia University Medical Center	University of Arizona
Cook Children’s Hospital	University of California Los Angeles
Dana Farber/Boston Children’s Hospital	University of Colorado, Anschutz Medical Campus
Duke Children’s Hospital & Health Center	University of Florida Health
Helen DeVos Children’s Hospital	University of Minnesota Medical School
Hospital for Sick Children	University of Texas Southwestern Medical Center
John Hopkins All Children’s Hospital	University of Wisconsin
Johns Hopkins Sidney Kimmel Comprehensive Cancer Center	Winship Cancer Institute, Emory
Lucile Packard Children’s Hospital Stanford	Yale University and Yale New Haven Children’s Hospital

**Figure 1:**
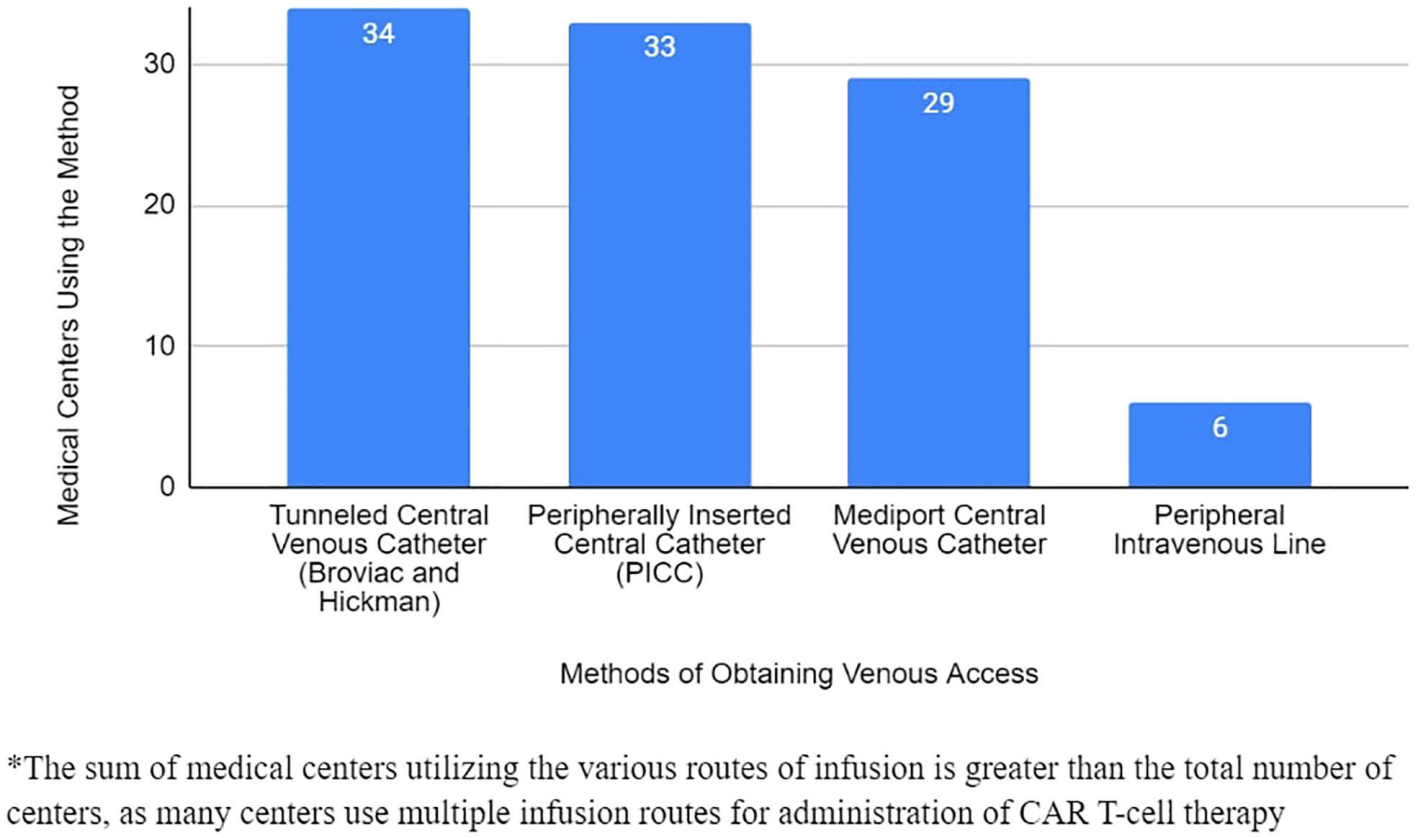
Venous Access Methods for Delivery of CAR T-Cell Therapy Utilized by PRWCC Medical Centers.

All 34 responding sites reported using tunneled central venous catheters, including Broviac^®^ and Hickman^®^ lines, as accepted standard line options for CAR T cell infusion. Use of mediports as a standard clinical practice was reported in 29 of 34 sites (85%). Of the sites using mediports, there were no incidences of CAR T cell therapy infiltration reported. One site reported exclusive use of mediports for CAR T cell therapy infusion. Notably, 6 of 34 centers (18%) also reported using peripheral intravenous lines for administration of CAR T cell therapy (**Figure 1**). Sites using peripheral intravenous catheters reported no incidences of CAR T cell therapy infiltration.

**Figure 2:**
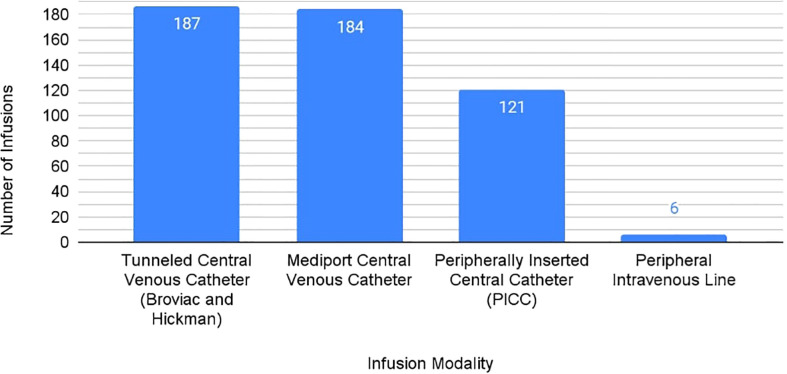
Rates of Infusion Modalities Across PRWCC Sites for CAR T-cell Therapy Administration.

From responding PRWCC sites, 489 patients with a total of 504 infusions were reported with known route of CAR T cell therapy administration. Of these infusions, 187 were administered by central venous catheter (37%), 184 by mediport (37%), 121 by PICC (24%), and 6 with peripheral intravenous catheter (1%) (**Figure 2**).

## Discussion

Limited data exists describing accepted infusion route for CAR T cell therapy. The paradigmatic use of cell therapy with greatest clinical experience is hematopoietic cell therapy, where practice often avoids mediport use due to high complexity of cell procurement and risk of cell infiltration. In the early days of CAR T cell commercialization, similar practice was adopted by select sites, however, we hypothesized that mediport usage for CAR T cell infusion has since been adopted as a safe accepted practice, with minimal risk of infiltration. Using the PRWCC framework, we conducted a survey of clinical practice for line usage for administration of CAR T cell therapy in children and young adults with B cell malignancies and infiltration rates. We administered a follow-up survey to quantify patient volume infused using different routes of administration. Of the medical centers surveyed, 29 of 34 sites reported routine use of mediports, indicating that mediports are a commonly utilized method of venous access for CAR T cell therapy infusion.

Across surveyed medical centers, 184 CAR T cell products were infused using mediports with no reported instances of infiltration. These findings, which represent clinical practice from Tisagenlecleucel FDA approval in 2017 through September 1^st^, 2022, suggest that concerns for mediport dislodgement and cell infiltration have not emerged as a clinical challenge. Furthermore, prior studies comparing mediport and PICC line usage for chemotherapy administration in pediatric and adult patients with solid malignancies have found distinct advantages with mediport usage (12, 13). These studies demonstrated fewer post-treatment complications (thrombosis, infection) and improved quality of life with mediport usage, when compared to PICC lines (12, 13). Medical centers have also found increased ease of access and reduced number of needle sticks with mediports (10, 11). These results exhibit the clinical advantages of mediports when compared to other methods of venous access. In the context of CAR T cell therapy, an additional consideration in utilizing a central line is to ensure patients have readily available access in the event that they experience high grade toxicities. Together, these findings support the use of mediports already in place for CAR T cell infusion, as opposed to the placement of a new PICC line, a procedure often requiring anesthesia in pediatric patients, for this purpose.

While our study highlights real-world use of mediports for the delivery of CAR T cell therapy, the possibility of mediport infiltration remains. We therefore highlight the importance of using standard operating procedures for mediport infusions, including appropriate training prior to access and confirmation of blood return prior to infusing the cellular therapy product.

In conclusion, to our knowledge, we report the first multi-institutional reporting (N=489) on routes of commercial CAR T cell delivery across children and young adults. We describe that current clinical practice in medical centers across the United States indicates that mediports are a commonly utilized method of CAR T cell therapy administration. Of centers that use mediports, 184 patients had CAR T cells delivered using mediports, with no reported instances of therapy infiltration. From these clinically based findings, we can draw support for the obviation of new PICC line insertion for CAR T cell infusion in patients who already have mediports in place. These results support the usage of mediports as a safe, accepted standard line option for the infusion of CAR T cell therapy.

## Data availability statement

The original contributions presented in the study are included in the article/**Supplementary Material**. Further inquiries can be directed to the corresponding author.

## Author contributions

ME and LS contributed to conception, design, statistical analysis, and writing of the manuscript. MK and DB contributed to database organization and statistical analysis. SP, SJ, KC, CE, NK, CP, PS, MH, ES, SB, JT, MM, JR, CLB, GM, RR, KT, TD, EK, DBS, DS, SO, CC, HP, TP, NS, VH, JS, TL, EF, KM, TQ, JK, JL, VF, and CB contributed to data collection and writing of sections of the manuscript. All authors contributed to the article and approved the submitted version.

## Supplementary material

Supplementary Figure 1 Survey #1.

Supplementary Figure 2 Survey #2.
