# Supplementary material for: Mediport use as an acceptable standard for CAR T cell infusion
Source: Front Immunol. 2023 Oct 27;14:1239132. doi: 10.3389/fimmu.2023.1239132 (PMC10642031; doi:10.3389/fimmu.2023.1239132)
Supplement: Supplementary Figure 1 — Survey #1. [file Image_1.pdf]

Supplementary Figure 1: Survey 1 Distributed to PRWCC Medical Centers

1. Do you use mediports for the infusion of CAR T-cell therapy? Yes [ ] No []

Do you use peripheral IVs for the infusion of CAR T-cell therapy? Yes [] No [ ]

Do you use broviacs/hickmans for the infusion of CAR T-cell therapy? Yes [] No [ ]

Do you use PICCs for the infusion of CAR T-cell therapy? Yes [] No [ ]

2. Have you ever had a line infiltration when infusing CAR T-cell therapy

Using mediports? Yes [ ] No [ ] N/A [] If yes, explain:

Using peripheral IV? Yes [ ] No [] N/A [ ] If yes, explain:

Supplemental Figure 2: Survey 2 Distributed to PRWCC Medical Centers

Site: ______________________________

Total Patients infused with Commercial CAR from Aug 2017 – September 1, 2022: _____________

Route of Infusion	Mediport	Central Line	PICC	Peripheral IV
Patient Volume				
